# Gut microbiota and diet in colorectal cancer: Converging determinants of carcinogenesis

**DOI:** 10.1080/19490976.2026.2664684

**Published:** 2026-05-04

**Authors:** Bhupesh Kumar Thakur, Saurav Roy Choudhury, Williams Turpin, Alberto Martin

**Affiliations:** a Department of Immunology, University of Toronto, Toronto, Ontario, Canada; b Zane Cohen Centre for Digestive Diseases, Mount Sinai Hospital, Toronto, Ontario, Canada; c Department of Nutritional Sciences, Temerty Faculty of Medicine, University of Toronto, Ontario, Ontario, Canada

**Keywords:** Colorectal cancer, gut microbiota, diet, *pks^+^* *E. coli*, inflammation

## Abstract

Diet and the gut microbiome are major, interdependent determinants of colorectal cancer (CRC) risk. This review discusses current evidence on how dietary patterns reshape microbial ecology, modulate microbial virulence, and alter host metabolic, inflammatory, and oncogenic pathways to influence colorectal carcinogenesis. We highlight key CRC-associated microbes, including *pks⁺ Escherichia coli*, *Fusobacterium nucleatum*, enterotoxigenic *Bacteroides fragilis*, and *Streptococcus gallolyticus*, and discuss how diet governs their abundance, toxin production, and oncogenic potential. Mechanistic investigations into diet-microbe interactions reveal how pro-inflammatory, low-fiber Western-style diets foster mucosal inflammation, generation of reactive oxygen and nitrogen species, and genotoxic microbial niches, whereas fiber- and polyphenol-rich diets support protective commensals and production of anti-inflammatory metabolites. We also outline major challenges, including interindividual microbiome variability and limited translational models, and propose future directions for integrating dietary, microbial, and host-targeted strategies for CRC prevention and therapy.

## Introduction

Colorectal cancer (CRC) is a major global health challenge, ranking third in incidence and second in cancer-related mortality worldwide. According to GLOBOCAN 2020, an estimated 1.9 million new CRC cases and 930,000 deaths occurred globally in 2020, with projections rising to 3.2 million cases and 1.6 million deaths annually by 2040 if current trajectories persist.^1^ Although CRC incidence remains highest in high-income regions, the most rapid increases are now observed in low- and middle-income countries, reflecting accelerating adoption of westernized lifestyles and dietary habits. A particularly concerning trend is the worldwide surge in early-onset CRC (in individuals who are <50 y), which also mirrors global shifts toward Western-style diets.^2-5^ Accumulating evidence have identified multiple dietary and host-related factors in modulating CRC risk. High consumption of red and processed meats, excessive alcohol intake, obesity, and chronic inflammatory conditions such as inflammatory bowel disease (IBD), along with underlying genetic susceptibility, are all associated with increased CRC risk.^6-12^ In contrast, several exposures have demonstrated protective effects, including regular aspirin use, higher circulating vitamin D levels, adequate folate intake, a diet rich in fiber, and greater physical activity.^10^ Together, these findings underscore the complex interplay between diet, host biology, and environmental factors in shaping CRC susceptibility.^4^^,^^13^ Mechanistically, these CRC risk-associated factors not only drive metabolic dysfunction and systemic inflammation but also disrupt gut microbial composition throughout life. Altered microbial communities, characterized by loss of beneficial fiber-fermenting taxa and expansion of pro-inflammatory, genotoxic, or metabolically adverse species, interact with the colonic epithelium to promote DNA damage, epigenetic reprogramming, immune dysregulation, and impaired barrier integrity. Collectively, these environmental and dietary perturbations create a microbiota-driven protumorigenic intestinal microenvironment that accelerates CRC initiation and progression.

CRC develops through the gradual accumulation of somatic mutations in key oncogenes and tumor suppressor genes within colonic stem cells. ^14^, ^15^ This process follows the classical adenoma‒carcinoma sequence, in which normal colonic epithelium sequentially progresses to adenomatous lesions and ultimately to invasive CRC. This sequence is driven by the accumulation of genetic and epigenetic alterations, including mutations in *APC*, *KRAS*, *TP53*, and DNA mismatch repair genes, accompanied by aberrant activation of signaling pathways such as Wnt/β-catenin and MAPK. However, the carcinogenic mechanisms underlying colitis-associated CRC differ from the classical adenoma‒carcinoma sequence and instead follow the inflammation-dysplasia-carcinoma pathway. In the classical adenoma‒carcinoma sequence, *APC* mutations typically appear first, followed by *KRAS* mutations, with *TP53* mutations arising at later stages of tumor progression. In contrast, colitis-associated CRC is characterized by early *TP53* mutations, followed by *APC* and *KRAS* mutations at later stages, reflecting an inflammation-driven and temporally distinct mutational trajectory.^16^, ^17^ These carcinogenic pathways can be accelerated by chronic inflammation, oxidative stress, and exposure to genotoxins, which induce DNA damage, promote epithelial proliferation, and alter local immune responses. Increasing evidence further implicates gut microbial dysbiosis as a key modulator of this neoplastic trajectory.^18^ Thus, the adenoma‒carcinoma sequence provides a biological framework linking environmental exposures, microbial perturbations, and molecular alterations in the stepwise evolution of CRC.^14^, ^19-21^

Interactions between environmental exposures, particularly diet, and the gut microbiota determine the outcome of many biological processes in health and disease, including cancer. Moreover, diet profoundly shapes the composition and metabolic output of the gut microbiota, thereby influencing CRC risk.^21-23^ Western-style diets (high-sugar, high-fat with low fiber content) or low-carbohydrate, low-fiber diet, in particular, have been shown to induce gut dysbiosis characterized by the expansion of pathogenic or bile acid-tolerant strains and depletion of short-chain fatty acids (SCFAs)-producing microbes. Such microbial and metabolic disturbances can compromise epithelial barrier integrity, trigger mucosal inflammation, and activate oncogenic signaling cascades,^24-26^ collectively expediting the adenoma‒carcinoma sequence even at a younger age.^2^^,^^3^^,^^5^ Deciphering how specific dietary components and microbial dysbiosis converge to drive colorectal carcinogenesis is therefore crucial for developing microbiota-targeted preventive and therapeutic interventions.

In this review, we provide a comprehensive overview of the principal microbial taxa linked to CRC, and discuss the dietary factors known to influence their prevalence, activity, or pathogenic potential. We further integrate emerging mechanistic evidence illustrating how interactions between diet and the gut microbiota remodel the intestinal and tumor microenvironment, focusing on the ways in which diet-derived metabolites (such as secondary bile acids, heme, and sulfur compounds) and microbially produced molecules (including SCFAs and genotoxic agents) collectively drive chronic inflammation, disruption of epithelial barrier integrity, and genomic instability. Finally, we discuss the key challenges, such as interindividual microbial heterogeneity, dynamic dietary patterns, confounding host factors, and limitations of current multiomics tools, that complicate the interpretation of the complex and bidirectional relationship between the gut microbiome and diet in CRC pathogenesis.

### The dysbiotic gut microbiome in CRC

Microbial density rises along the gastrointestinal tract and peaks in the colon, which contains ~10¹² bacteria per gram of luminal content.^27^ Consequently, colorectal tumors harbor some of the highest microbial biomass among human cancers.^28^ Human studies consistently show that the gut microbiome of individuals with CRC differs markedly from that of healthy individuals, exhibiting reduced levels of beneficial commensals and an overrepresentation of potentially pathogenic taxa in both fecal ^29,30,31^ and mucosal samples.^32^^,^^33^ These alterations in gut microbiome are strongly linked to disrupted epithelial barrier function, increased intestinal permeability, elevated circulating lipopolysaccharide levels, and chronic low-grade inflammation, collectively fostering a protumorigenic colonic environment. A large multicohort meta-analysis published in 2019 identified 29 fecal microbial species consistently enriched in CRC across geographically diverse populations, establishing globally generalizable taxonomic and functional microbiome signatures of CRC.^31^ Although specific bacterial taxa are reproducibly enriched in CRC tissues, and their presence correlates with adverse clinical outcomes,^31^ establishing a causal relationship remains challenging. CRC develops over many years prior to diagnosis, and the absence of CRC-associated microbes in advanced tumors does not exclude their involvement during earlier stages of tumor initiation. Nevertheless, recent preclinical research supports the procarcinogenic role of CRC-associated microbes, demonstrating their ability to actively promote tumor initiation, growth, and dissemination.^19^^,^^23^^,^^34-40^

In parallel, fecal and mucosal microbiome profiles in CRC frequently display a substantial reduction in health-promoting microbes, particularly *Lactobacillus* species and major butyrate-producing genera such as *Lachnospiraceae.*^5^, ^27^, ^41^ This loss of protective microbes and microbial functions reflects a shift away from a homeostatic gut community to one that allows opportunistic and protumorigenic microbes to expand. This microbial imbalance may compromise epithelial resilience and SCFAs production while amplifying inflammatory signaling, oxidative stress, and genotoxic pressures, conditions that collectively facilitate malignant transformation and tumor progression.^5^ Thus, evaluation of the microbiome in CRC risk requires considering not only the enrichment of pathogenic or procarcinogenic bacteria but also the depletion of beneficial commensal microbes that normally help reinforce resistance to carcinogenesis. Indeed, species such as *Faecalibaculum rodentium* and its human homolog *Holdemanella biformis* protect against the development of intestinal tumors and exert antitumor effects through the production of butyrate, a SCFA that suppresses NF-κB signaling and reduces proinflammatory cytokines, including IL-6 and TNF.^42^ Moreover, fasting-mimicking diets suppress colorectal tumorigenesis in part by enriching *Bifidobacterium pseudolongum* in the feces, which produces L-arginine that promotes the differentiation of CD8⁺ T cells into tissue-resident memory cells, thereby enhancing antitumor immunity.^43^ However, despite increasing recognition of the protective functions of these beneficial commensals, the mechanistic consequences of their depletion in CRC development remain inadequately defined and require more systematic investigation. Altogether, these findings highlight the dual roles of the gut microbiome, both as a source of carcinogenic stimuli and as a reservoir of protective metabolites, and underscore the importance of understanding microbe‒host interactions in CRC development to identify potential strategies for microbiota-targeted CRC prevention and therapy.

### Pro-tumorigenic microbes in CRC

Many microbes have been found to be associated with tumors, but might not be cancer drivers and are associated with CRC lesions due to the altered environment provided by the tumor. However, several taxa have been consistently implicated in CRC risk, each employing distinct yet sometimes converging mechanisms that contribute to tumor initiation, progression, or metastatic dissemination. Among the most extensively studied are *pks*^*+*^ *Escherichia coli*, *Bacteroides fragilis*, *Fusobacterium nucleatum*, *Streptococcus gallolyticus*, *Peptostreptococcus anaerobius, Clostridioides difficile,* and *Campylobacter jejuni.*^19^^,^^23^^,^^34^^,^^36^^,^^37^^,^^39^^,^^44-49^ These microbes promote CRC through a broad spectrum of tumor-promoting mechanisms, including the production of genotoxins that induce DNA damage and mutations in host epithelial cells,^35^, ^38^, ^40^, ^50^, ^51^, ^52^ modulation of epithelial signaling pathways, alteration of mucosal immune responses, and the establishment of chronic proinflammatory states. Collectively, these divergent mechanisms by which these microbes promote CRC underscore the multifactorial nature of colorectal tumorigenesis. Moreover, these microbes often function within complex polymicrobial biofilms in the gut, thereby amplifying one another's carcinogenic potential. Their individual and cooperative roles in CRC development are discussed in detail in the following sections.

### Pks^+^ Escherichia coli

*E. coli* is a gram-negative, facultative anaerobe and one of the earliest colonizers of the human gut. Although a vast majority of the identified *E. coli* strains are harmless commensals, certain strains, particularly classified as phylogroups B2, harbor virulence factors and cause intestinal and extraintestinal diseases, including CRC. Strains within this phylogroup frequently carry the *polyketide synthase* (*pks*) pathogenicity island, which encodes the biosynthetic machinery for colibactin, a genotoxic hybrid polyketide-nonribosomal peptide that can induce DNA damage and mutations.^35^, ^51^, ^53^, ^54^, ^55^, ^56^ Colibactin has been strongly linked to colorectal carcinogenesis; however, its primary evolutionary function is thought to be interbacterial competition rather than host mutagenesis. Supporting this notion, we and others have shown that colibactin production substantially alters the gut microbial community under both disease-free and diseased conditions.^23^, ^57^, ^58^ However, how these community shifts translate into a selective ecological advantage for colibactin-producing strains within the gut remains unclear and warrants further investigation.

*Pks⁺* *E. coli* induces DNA damage in host cells in a contact-dependent manner, requiring adhesin-mediated binding to epithelial cells,^59^, ^60^ and triggers cytopathic effects in epithelial cells, including G2/M cell-cycle arrest, cell elongation, and premature senescence.^23^, ^61^, ^62^ These effects are mediated by cyclomodulins, a family of bacterial effectors that cause DNA damage. Among these, colibactin is the best characterized, which promotes colon tumorigenesis by alkylating DNA, forming DNA adducts, interstrand crosslinks, or double-strand breaks, and causing mutations in *APC* to stabilize *β*-catenin and prolonged activation of the Wnt signaling pathway.^35^, ^51^, ^52^, ^54^, ^55^, ^56^ Colibactin-induced DNA lesions are often incompletely repaired, increasing mutational burden and promoting malignant transformation. Recent studies have elucidated colibactin's biosynthesis and mutational consequences. The *pks* locus carries 19 *clb* genes, encoding key proteins such as ClbA (phosphopantetheinyl transferase), ClbM (transporter), and ClbP (peptidase required for final toxin maturation), involved in colibactin assembly and maturation.^63^ The reactive cyclopropane warhead in colibactin enables DNA alkylation that results in DNA adducts and likely drives the mutational signature identified for colibactin.^54^, ^56^ Seminal studies investigating colibactin-induced mutations identified a distinct mutational signature associated with colibactin i.e. single-base substitutions (SBS-*pks)*, and a small indel (ID-*pks*) with deletions and insertions at T sites.^35^, ^50^ These signatures, designated SBS88 and ID18, have since been detected in several cancer tissues, including CRC tumors, highlighting the clinical relevance of colibactin in CRC development.^35^, ^51^ Moreover, *pks⁺* *E. coli* infection or exposure to synthetic colibactin has been shown to exacerbate mutations characteristic of mismatch-repair deficiency (MMRd), suggesting that colibactin either contributes to the mutational landscape of MMRd tumors or that the MMR pathway is a primary mechanism for repairing colibactin-induced DNA lesions.^64^ In support of the latter notion, MMR-deficient mice are more susceptible to colibactin-dependent colon tumorigenesis.^23^ Thus, elucidating the biosynthetic pathway of colibactin and the mechanisms regulating its expression is of considerable interest, as pharmacologic inhibition of this toxin or its upstream regulators may offer avenues for CRC prevention or therapy, though such strategies face substantial challenges given the decades-long timeline of CRC development.

The colibactin-associated signatures, SBS88 and ID18, are significantly enriched in early-onset colorectal cancer, occurring approximately 3.3-fold more frequently in individuals diagnosed before 40 y of age compared with those diagnosed after 70 y.^65^ Consistently, *pks⁺ E. coli* commonly colonizes the gut early in life,^66^ and colibactin-associated mutations accrue slowly over decades, paralleling the long natural history of sporadic CRC. This raises the possibility that persistent colonization or episodic activation of the *pks* island contributes cumulatively to tumorigenesis. Consistently, we and others have reported that bacterial abundance, timing and magnitude of colibactin exposure, inflammatory milieu, and interactions with competing microbes are the key factors that influence the oncogenic impact of *pks*^*+*^ *E. coli*.^23^, ^36^, ^59^, ^60^ Thus, defining the environmental cues, including dietary factors, their metabolites, and inflammatory signals, that promote the expansion, persistence, adhesion to the epithelium, or virulence activation of *pks⁺ E. coli* is essential for uncovering modifiable factors that shape long-term CRC risk. These determinants and their mechanistic implications in CRC are examined in detail in the following section.

### Bacteroides fragilis

*B. Fragilis* is a gram-negative anaerobic commensal and an early gut colonizer and has been reported to play an important role in T-cell-mediated immune homeostasis. Although it typically represents only 1%–2% of cultured fecal bacteria, it is a common member of the human gut microbiota.^67^, ^68^, ^69^ This species has been divided into two types based on their production of a zinc-dependent metalloprotease, *B. fragilis* toxin (BFT): nontoxigenic strains (NTBF) do not produce the toxin, whereas enterotoxigenic strains (ETBF) secrete three isoforms of BFT (BFT-1–3). ETBF has been detected in the mucosa of CRC patients. Moreover, its detection in colonic mucosa is associated with neoplasia, especially in late-stage CRC, and is also associated with a poorer prognosis.^70^ Preclinical studies have found that BFT drives oncogenesis by cleaving the extracellular domain of E-cadherin, disrupting epithelial integrity, and inducing spermine oxidase, which alters cellular morphology and generates oxidative stress. Cleavage of E-cadherin also disrupts epithelial integrity, causes cytosolic accumulation of *β*-catenin, and promotes its nuclear translocation, where it binds TCF/LEF transcription factors to upregulate proto-oncogenes such as c-MYC and cyclin D1 (CCND1). Elevated ETBF levels also correlate with increased AXIN and CTNNB1 expression in tumor tissues. BFT further enhances MAPK and WNT signaling and stimulates proinflammatory cytokines, including IL-8, promoting a tumor-permissive environment.^46^, ^71^, ^72^, ^73^ Moreover, ETBF upregulates JmjC-domain containing histone demethylase 2B (JMJD2B) levels in a TLR4-NFAT5-dependent pathway, and promotes colonic stemness to CRC development.^74^ In *Apc*^*Min/+*^ mice, ETBF induces a proinflammatory tumor microenvironment via STAT3, TH17, and γδ T cell-derived IL-17, with TH17 cells amplifying IL-17-driven tumorigenesis.^37^ Despite its profound effects on epithelial signaling, inflammation, and CRC development, ETBF does not induce a distinct mutational signature in CRC tumors,^75^ suggesting that this bacterium produces carcinogenesis independent of direct genotoxicity. Importantly, BFT expression is influenced by several environmental cues and dietary factors that can influence the tumorigenic potential of this bacterium, and is further explored in the following section.^76^

### Fusobacterium nucleatum

*F. nucleatum* is a gram-negative anaerobe predominantly found in the oral cavity and rarely colonizes the healthy lower gastrointestinal tract.^77^, ^78^ However, it is found in high abundance in CRC tumors compared to the adjacent normal tissue, and its abundance correlates with disease recurrence, metastasis, and poor prognosis.^78^, ^79^, ^80^, ^81^ The mucosal abundance of *F. nucleatum* varies among CRC patients, suggesting that individual-specific effector factors may regulate its abundance. CRC tumors express high levels of d-galactose-β(1-3)-*N*-acetyl-d-galactosamine (Gal-GalNAc) sugar moieties at early and metastatic stages of disease. *F. nucleatum* adheres to tumor cells via Fap2 and FadA adhesins, binding Gal-GalNAc and E-cadherin, respectively.^44^, ^82^ Colon tissues from CRC patients exhibit 10–100-fold higher *fadA* levels relative to healthy individuals, and this elevation corresponds to heightened oncogenic and inflammatory gene expression.^44^ FadA-E-cadherin interaction triggers *β*-catenin/Wnt signaling, promoting c-MYC and cyclin D1 expression, while Fap2 binds the immune checkpoint TIGIT, suppressing NK and T-cell activity.^44^, ^82^ It also binds DHX15, an RNA helicase family protein expressed on colorectal tumor cells, and activates ERK/STAT3 signaling, drives NF-κB–mediated inflammation, IL-17–rich tumor microenvironments, and chemoresistance via autophagy induction.^83^, ^84^ It has also been noted that *F. nucleatum* induces epigenetic changes, increases DNA methyltransferase activity and causes hypermethylation of tumor suppressor genes. This process also contributes to a high microsatellite instability (MSI-H) and a CpG island methylator phenotype (CIMP) in CRC, and impairs Chk2 signaling, disrupting cell cycle regulation and DNA repair.^85^, ^86^ Colonization of *F. nucleatum* in mouse models elevates the SCFA and formate levels, having immunomodulatory potential, can modulate immune responses and tumor metabolism via GPCR and aryl hydrocarbon receptor (AhR) pathways, respectively, promoting cancer stemness and glutamine-dependent growth.^87^, ^88^ *F. nucleatum*, in contrast to the other CRC-associated microbes, doesn’t produce any toxins, but consistently emerges as a CRC-enriched microbe and promotes tumorigenesis in multiple preclinical models through its ability to shape a protumorigenic and prometastatic microenvironment through modulation of host immunity and induction of metabolic reprogramming. However, the extent to which *F. nucleatum*-induced CRC development is influenced by environmental cues, including dietary factors, remains incompletely understood and is discussed in the following section.

### *Campylobacter* *spp*

*Campylobacter* is a gram-negative bacterium that is enriched in CRC lesions compared to adjacent normal tissue.^33^, ^89^ This bacterium produces cytolethal distending toxin (CDT), a genotoxin capable of inducing DNA double-strand breaks and promoting intestinal tumorigenesis and metastasis in preclinical models of CRC.^90^, ^91^ Beyond CDT-mediated genotoxicity, several Campylobacter species can activate oncogenic signaling pathways, including mTOR and *β*-catenin, and stimulate epithelial cell proliferation. These bacteria also exacerbate inflammatory responses, potentially generating mucosal conditions that facilitate the expansion of other CRC-associated microbes such as *F. nucleatum*. Meta-transcriptomic profiling of human CRC tissues has found co-aggregation of *Campylobacter* *spp.*, particularly *Campylobacter concisus*, with *Fusobacterium* species within tumor-associated biofilms.^92^ This spatial colocalization highlights the possible ecological or functional cooperation between these taxa within the tumor microenvironment, which may collectively enhance microbial persistence and tumor-promoting activities.

### Streptococcus gallolyticus

*S. gallolyticus* subsp. *gallolyticus* (*Sgg*) has emerged as a clinically significant onco-microbe due to its capacity to enhance epithelial proliferation and accelerate colorectal tumorigenesis.^93^ Not all identified strains behave uniformly, as strain TX20005 robustly drives epithelial proliferation, whereas strain ATCC 43143 lacks this activity. A key genomic determinant of *Sgg* pathogenicity is the *Sgg* pathogenicity-associated region (SPAR). Loss of this locus compromises their ability to colonize the colon, adhere to epithelial surfaces, activate host proliferative pathways, and promote tumor growth *in vivo.*^93^ *Sgg* also encodes a bile salt hydrolase and produces a bacteriocin, gallocin, whose activity is markedly enhanced in the presence of bile acids. These features provide *Sgg* with a selective growth advantage in the bile acid-enriched environment of adenomatous colorectal tissue, supporting its persistence and competitive dominance within the evolving tumor niche.^94^ Clinically, *Sgg* bacteremia is strongly linked with underlying colorectal neoplasia.^47^ Patients with *Sgg* bloodstream infections exhibit an approximately 60% increased likelihood of harboring adenomas or adenocarcinomas, and nearly half of individuals with *Sgg*-associated endocarditis develop colorectal tumors within 5 y.^47^, ^95^ Mechanistic studies show that *Sgg* activates multiple oncogenic pathways, including c-Myc, Wnt/β-catenin, and proliferating cell nuclear antigen (PCNA).^95^ Emerging evidence indicates that *Sgg* can activate the aryl hydrocarbon receptor (AhR) and downstream CYP1 family enzymes in CRC cells. This activation enhances the biotransformation of environmental and endogenous toxic substrates, generating reactive intermediates capable of forming DNA adducts. Such metabolically driven genotoxic stress may further accelerate CRC progression.^94^

### Peptostreptococcus anaerobius

*P. anaerobius*, a gram-positive anaerobic bacterium, is found enriched in both mucosa and feces of patients with CRC.^29^, ^96^ Moreover, colonization with *P. anaerobius* accelerates intestinal tumor formation in a mouse model of CRC.^48^, ^96^ Mechanistic work has identified a bacterial surface protein, putative cell wall binding repeat 2 (PCWBR2), as a ligand responsible for engaging the α2β1 integrin receptor expressed on CRC epithelial cells. PCWBR2 binding to α2β1 integrin triggers recruitment and activation of Src-family kinases, leading to phosphorylation of focal adhesion kinase (FAK) and subsequent activation of PI3K-AKT signaling. These pathways collectively enhance epithelial proliferation, stimulate NF-κB activation, and activate inflammatory and prosurvival programs.^48^ *P. anaerobius* further amplifies tumorigenic processes through innate immune signaling. Activation of TLR2 and TLR4 increases intracellular reactive oxygen species (ROS), creating oxidative stress that fuels epithelial proliferation. This ROS-dependent response is closely linked to upregulation of cholesterol biosynthesis, a metabolic program essential for membrane production, cell growth, and survival.^97^ Beyond direct epithelial signaling, *P. anaerobius* also reshapes the tumor immune microenvironment by increasing the tumor infiltration of myeloid-derived suppressor cells (MDSCs), which are known to impair antitumor cytotoxic CD8⁺ T-cell responses, and thus can promote angiogenesis and metastatic progression.^48^ Together, existing studies highlight the multifaceted pathogenicity of *P. anaerobius*. By concurrently modulating epithelial oncogenic pathways, innate immune signaling, redox balance, host lipid metabolism, and local immunosuppression, this microbe influences several canonical hallmarks of cancer, including genome instability, metabolic reprogramming, and evasion of antitumor immunity.

### Clostridioides difficile

*Clostridioides difficile*, previously known as *Clostridium difficile*, is a Gram-positive, spore-forming anaerobe that predominantly colonizes the colon and is generally detected at low abundance in fecal gut microbiota of healthy individuals.^98^ However, broad-spectrum antibiotic exposures facilitate the colonic expansion of resistant *C. difficile* strains and the development of antibiotic-associated diarrhea.^98^, ^99^ Virulent strains secrete the *C. difficile* toxin TcdA and TcdB, which disrupt epithelial barrier integrity, induce cell death, and elicit strong inflammatory responses, giving rise to diarrhea and colitis in susceptible individuals.^99^, ^100^ Increasing evidence implicates *C. difficile* in CRC development, with elevated abundance observed in tumor tissues compared with adjacent nonmalignant mucosa.^101^ In preclinical murine models, polymicrobial biofilm communities derived from CRC patient-derived mucosal slurries promote colonic tumor formation; notably, selective depletion of *C. difficile* from these communities abolishes their tumorigenic potential, demonstrating that *C. difficile* is both necessary and sufficient to convert a nontumorigenic microbial consortium into a pro-tumorigenic one, at least in a mouse model.^40^ Mechanistically, TcdB induces Wnt/β-catenin pathway in colonic epithelial progenitor cells, promotes ROS production, and drives the expansion of protumorigenic IL-17-producing immune cells.^40^ In addition, TcdB has been shown to induce cellular senescence and the senescence-associated secretory phenotype (SASP), which may facilitate the progression of preneoplastic lesions to overt CRC.^100^ However, direct evidence linking *C. difficile* to human CRC remains limited, and its role in CRC initiation or progression therefore warrants further investigation.

## Microbial cooperation in CRC development

While substantial progress has been made in elucidating the roles of individual microbes in CRC pathogenesis, emerging evidence suggests that the collective organization and functional capacity of microbial communities may exert a stronger influence on CRC risk and progression by jointly reshaping the metabolic, immune, and inflammatory landscape of the colon. The synergistic microbial communities often organize into biofilms, where close microbial proximity amplifies oncogenic signals, heightens inflammatory and genotoxic stress, and shapes a microenvironment that supports CRC initiation and progression.^38^, ^102^ ETBF frequently colocalizes with *pks*⁺ *E. coli* in mucosal biofilms, particularly in patients with familial adenomatous polyposis, and enhances *pks*⁺ *E. coli*-driven tumorigenesis by fostering an IL-17-rich inflammatory milieu.^38^ Likewise, *Fusobacterium* consistently occupies the same biofilm niches as *Campylobacter* species, especially *Campylobacter concisus*, in human CRC tissues;^92^ however, whether these microbes functionally coordinate to drive tumorigenesis remains unclear. Biofilm formation provides a key mechanism by which bacteria collectively secure and stabilize their foothold in the tumor microenvironment. This polymicrobial community in intestinal biofilms erodes the mucus layer, increases epithelial permeability, and delivers concentrated genotoxins and inflammatory stimuli directly to host cells, to produce genotoxicity and promote carcinogenesis. Within polymicrobial communities, bacteria communicate with each other and with the host through quorum sensing, a chemical signaling system that coordinates collective behaviors in a density-dependent manner. Quorum sensing relies on the production, release, and detection of diffusible autoinducer molecules, enabling microbial populations to assess cell density and synchronize gene expression across intra- and interspecies networks. This coordinated signaling supports community-level functions that are inefficient at the single-cell level. Growing evidence indicates that quorum-sensing-regulated activities influence multiple aspects of tumor biology, including biofilm organization, virulence factor expression, immune modulation, and cancer progression and metastasis.^103^, ^104^ Together, these findings indicate that ecological and functional cooperation within microbial communities promotes their persistence and tumorigenic activity in the gut, effects that may be further shaped by environmental factors, including diet, and underscore the need for integrative approaches to understand community-driven CRC pathogenesis.

## Dietary factors influencing CRC risk

Diet represents one of the predominant etiologic factors associated with the initiation and progression of CRC. Seminal work by Doll and Peto (1981) estimated that ~35% of all cancer-related deaths, and up to 90% of gastrointestinal cancer mortality, could be attributed to dietary factors.^105^, ^106^ Since then, epidemiological studies over the past four decades have consolidated the strong association of dietary factors and lifestyle in CRC. More recently, dietary patterns have also been implicated as key drivers in the alarming rise in early-onset CRC among young adults.^107^, ^108^ Certain dietary exposures, particularly Western-style diets enriched in processed and red meats, highly refined grains/starch, and sugars, have been consistently associated with increased risk of CRC, whereas consumption of fish, poultry, yogurt, certain legumes, and other plant-based foods is associated with reduced CRC risk.^109^, ^110^ In the United States, a substantial proportion of CRC-related mortality is attributed to dietary patterns: low fiber intake (~10%), consumption of processed meats (~8%), red meat (~5%), and insufficient dietary calcium (~5%).^111^

**Figure 1.**
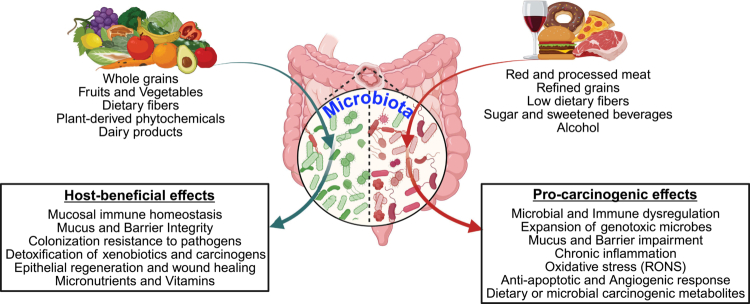
Diet shapes gut microbiota and colorectal cancer risk. Diets rich in fruits, vegetables, whole grains, fiber, and dairy support a beneficial microbiota, promoting immune homeostasis, barrier integrity, and detoxification, whereas diets high in red and processed meat, refined grains, sugar, and alcohol drive dysbiosis, chronic inflammation, barrier impairment, expansion of genotoxic pathogens, and production of carcinogenic metabolites. (Created with BioRender.com).

The influence of dietary factors on CRC risk is mediated, in part, through their ability to reshape the metabolic landscape of gut mucosa and tumor microenvironment, thereby impacting malignant transformation of epithelial cells, cancer cell metabolism, and tumor growth dynamics. Beyond direct effects on host tissues, dietary components are now recognized to profoundly modulate both the composition and functional activity of the gut microbiome,^112^, ^113^ further contributing to malignant transformation and cancer progression.^113^, ^114^ Certain dietary patterns, such as red and processed meats (e.g., beef, pork, and lamb), refined grains and sugars, and alcohol, promote chronic inflammation, genotoxic stress, and tumorigenic signaling and are therefore considered procarcinogenic. In contrast, diets rich in fiber, polyphenols, and omega-3 fatty acids exhibit anticarcinogenic properties by strengthening epithelial barrier function, reducing inflammation, and supporting the growth of beneficial microbial communities ^115^ (Figure 1). In the following sections, we examine dietary patterns consistently implicated in CRC development and emphasize how diet-derived metabolites, both those directly originating from dietary components and those generated through microbial metabolism, collectively shape either a tumor-permissive or tumor-protective colonic microenvironment.

### Red and processed meat

Red and processed meat remains as one of the well-established dietary risk factors for CRC. Epidemiological evidence consistently supports a link between meat consumption and CRC incidence in both men and women, with some studies suggesting a stronger association in men.^11^ It is estimated that for each 100 g/d increase in red and processed meat intake, the risk of CRC increases by 12%, and of distal CRC by 70%.^11^ Based on accumulating epidemiological and mechanistic evidence regarding the influence of diet in CRC, the International Agency for Research on Cancer (IARC) categorized red meat as ‘probably carcinogenic’ (Group 2A), while processed meat has been classified as carcinogenic in humans.^110^ The Western-style diets, typically high in red and processed meat, saturated fats, and refined carbohydrates, increase the risk of CRC by many folds.^116^ Building on this, recent studies have identified specific dietary components and meat-derived factors that contribute to this carcinogenic effect. Red meat is enriched in sulfur-containing amino acids and saturated fats, whereas processed meats contain inorganic sulfur used as preservatives; both features introduce dietary risk factors that promote the initiation of colonic adenomas.^117^, ^118^, ^119^ Multiple studies have linked high-sulfur diets to an increased incidence of early-onset colonic adenomas.^108^, ^109^ Additionally, heme iron, abundant in red meat, promotes the endogenous formation of *N*-nitroso compounds, potent gastrointestinal carcinogens through oxidative stress and colonocyte proliferation.^119^, ^120^, ^121^, ^122^ *N*-nitroso compounds are also a major group of compounds added to processed meats as preservatives. In addition, high-temperature cooking of meat generates heterocyclic amines and polycyclic aromatic hydrocarbons, both of which are established carcinogens in experimental models and are associated with CRC risk in humans.^123^, ^124^, ^125^, ^126^ These associations between meat-derived factors and CRC risk become even more pronounced in individuals with genetic predispositions, such as those with mutations in *APC* or *KRAS*.^122^, ^127^, ^128^ Individuals with higher activity of *N*-acetyltransferase enzymes (NAT1 and NAT2), potent enzymes for carcinogenic activation of heterocyclic amines, appear to have an increased CRC risk, although these associations require further validation.^129-131^

### Sugar and sweetened beverages

Beyond meat consumption, high intake of sugar and sugar-sweetened beverages (SSBs) substantially increases CRC risk. Epidemiological evidence shows that higher SSB consumption during childhood and adolescence is associated with a two-fold increase in CRC risk in adulthood among women.^132^ Moreover, the rapid rise in the consumption of SSBs, particularly beverages containing added sugars, high-fructose corn syrup, or sucrose, parallels increasing CRC incidence and mortality in young adults. Consistently, dietary glucose, fructose, and maltose have been associated with an increased risk of developing CRC in human studies.^133^^,^^134^ Mechanistically, a recent study supports this association by showing that glucose and fructose from SSBs can increase CRC metastasis through the regulation of sorbitol dehydrogenase.^135^ Furthermore, SSBs promote the formation of colorectal adenomas independent of obesity in genetically susceptible *Apc*^*min*^ mice, suggesting a direct tumor-promoting role of dietary sugars beyond their effect on weight gain.^136^ However, obesity remains an important overlapping factor. Refined sugar intake, often in combination with red and processed meat consumption, contributes to obesity, a well-established CRC risk factor. Overweight and obese individuals have an 18% and 32% greater risk of developing CRC, respectively.^137^^,^^138^ However, the observation that SSBs increase CRC risk even in the absence of obesity indicates that sugar-driven carcinogenesis may involve additional mechanisms, which remain incompletely defined and warrant further investigation.

### Alcohol

The International Agency for Research on Cancer (IARC) classifies alcoholic beverages as carcinogenic to humans, and alcohol consumption increases CRC risk by more than 50%.^12^^,^^110^^,^^139^ Among dietary factors, alcohol shows one of the strongest positive associations with CRC, with particularly robust links to rectal cancer and, to a lesser extent, proximal CRC. Although elevated alcohol intake increases the risk of colon and rectal cancers in both men and women, several studies indicate a stronger association with distal CRC compared to proximal CRC.^110^ Although the precise mechanism by which alcohol consumption promote CRC are not completely understood, studies have shown that the production of mutagenic compounds such as aldehydes (high concentrations) can impair DNA repair machinery, form DNA adducts, induce epigenetic alterations, promote lipid peroxidation, disrupt epithelial barrier integrity, dysregulate intestinal immune system, and promote reactive oxygen and nitrogen species (RONS), all of which could contribute to tumor initiation and progression.^140^ Additionally, alcohol interferes with one-carbon metabolism, particularly folate metabolism. Consistent with this, several studies have shown that adequate dietary folate intake helps reduce CRC risk, especially among individuals who consume alcohol.^141^^,^^142^ Moreover, ethanol promotes oxidative stress through ROS, a process that is involved in the pathogenesis of different types of cancers, including CRC.^143^^,^^144^ Altered insulin signaling, metabolic deterioration associated with poor diet, and impaired host immune defenses have been shown to be some of the other mechanistic pathways associated with alcohol-induced CRC incidence.

### Fruits and vegetables

A substantial body of evidence suggests that higher consumption of fruits and vegetables reduces CRC risk.^145^ Their protective effects are attributed to a range of anticarcinogenic constituents, including dietary fiber, antioxidants, B vitamins, minerals, and diverse phytochemicals. Stronger protective associations have been reported in specific subgroups, such as individuals with low red-meat intake, those who are obese or sedentary, and those who abstain from smoking or alcohol,^146-148^ although further studies are needed to make more definitive conclusions on the association of fruits and vegetables in CRC development or establish causal mechanisms.

### Whole grain

Burkitt's original hypotheses (1971) proposed that diets with refined whole grains with reduced dietary fiber could promote CRC, a concept that has since been supported by various epidemiological and mechanistic studies.^149-152^ Numerous studies report that higher whole-grain intake is associated with a significantly reduced risk of CRC. Whole grains are a rich source of vitamins, minerals, complex carbohydrates, and phytochemicals, many of which possess potential anticarcinogenic properties.^153^ The development of objective biomarkers of whole-grain intake (e.g., circulating alkylresorcinols) has helped address the inaccuracies in dietary reporting and processing variability.^154-156^ Analyses using these biomarkers show a strong inverse association between whole-grain intake and CRC risk, with the most pronounced protective effects observed for cancers arising in the distal colon.^157^

#### Dietary fibers

Fruits, vegetables, legumes, seeds, cereals, and nuts contain varying proportions of soluble and insoluble fibers, though no single food source provides all fiber types. Through the metabolic activity of gut commensals, these fibers are fermented into major SCFAs, including butyrate, propionate, and acetate.^25^ The role of dietary fiber in gut health has drawn substantial interest, as multiple studies demonstrate that low fiber intake is associated with increased risk of IBD and CRC.^158-161^ Dietary fiber can protect against CRC by increasing fecal bulk and water-holding capacity, accelerating intestinal transit, and thereby diluting and reducing the exposure of the colonic epithelium to microbial or dietary carcinogens.^162^ Moreover, a recent meta-analysis further reported that reduced fecal concentrations of butyrate, propionate, and acetate correlate with higher CRC incidence.^163^ Besides being the preferred energy source for colonocytes, a number of studies have shown the anti-inflammatory and anti-neoplastic properties of butyrate through its ability to reduce cellular proliferation to induce apoptosis. Under physiological conditions, butyrate is metabolized in mitochondria, and its lower concentration promotes the proliferation of colonocytes. However, butyrate acts as an HDAC inhibitor at higher concentrations and migrates to the nucleus instead of getting metabolized in the mitochondria. Since cancer cells utilize glycolysis as a primary energy source, a low concentration of butyrate enters the nucleus and inhibits the proliferation of neoplastic colonocytes by acting as an HDAC inhibitor.^164^ In addition, butyrate also signals through the regulatory T cell receptor GPR43, promoting Treg expansion, and thus contributing to its anti-inflammatory activity.^165^^,^^166^

Propionate, another major SCFA generated through microbial fermentation of dietary fiber, also exhibits antitumorigenic activity. Early studies demonstrated that propionate suppresses the proliferation of colorectal adenocarcinoma cells in part by inhibiting ornithine decarboxylase, a key enzyme in polyamine synthesis.^167^ More recent mechanistic work shows that propionate attenuates azoxymethane (AOM) plus dextran sulfate sodium (DSS)-induced colitis-associated CRC through signaling via the olfactory receptor OR51E2, leading to modulation of cAMP and MEK/ERK pathways.^168^ In addition, Ryu et al. reported that propionate suppresses CRC progression by targeting histone methyltransferase EHMT2 (also known as G9a) for HECTD2-mediated proteasomal degradation.^169^ Together, these studies highlight propionate as a key microbially derived metabolite that contributes to the anti-proliferative and anti-neoplastic effects of a fiber-rich diet.

In contrast, the role of acetate in colon carcinogenesis remains controversial. Some studies report acetate-mediated inhibition of proliferation in colon cancer cell lines, whereas others show minimal or no effect.^167^^,^^170-173^ Mechanistic work indicates that acetate can suppress CRC cell growth by disrupting mitochondrial and lysosomal homeostasis. Following uptake via monocarboxylate transporters, acetate impairs mitochondrial metabolism and activates a mitochondria-dependent apoptotic program. In parallel, acetate induces lysosomal membrane permeabilization and release of cathepsin D (CatD), which despite being frequently overexpressed in CRC, exerts an antiapoptotic function that partially blunts the cytotoxic effect of acetate.^170^ Taken together, these findings suggest that impact of acetate on CRC are highly context-dependent and may vary according to acetate concentration, receptor expression (e.g. GPR43/GPR41), metabolic state, tumor subtype, and differential engagement of mitochondrial–lysosomal stress pathways.

Overall, dietary fiber-derived SCFAs support epithelial barrier integrity, modulate immune function, and exert anti-inflammatory, antiproliferative, and antineoplastic effects. Collectively, these properties make dietary fiber a compelling preventive and therapeutic strategy for CRC.

#### Dairy products

Consumption of dairy and dairy-based products has been associated with a reduced risk of CRC, although evidence regarding the effects of specific dairy products, such as yogurt, cheese, whole or cultured milk, and skim milk, remains limited. Milk intake, in particular, has been linked to lower risks of distal colon and rectal cancers^151^^,^^174^ (https://www.wcrf.org/wp-content/uploads/2024/10/Colorectal-cancer-report.pdf). The beneficial effects of dairy-based products are thought to be due to the high calcium content and other micronutrients. Although calcium represents the central component in dairy-based protection from CRC, studies have identified other dairy constituents such as conjugated linoleic acid (CLA), a molecule with anti-inflammatory/immunomodulatory, antioxidant, and antineoplastic properties.^174-178^ In animal models, supplementation of CLA was associated with a lower risk of CRC.^176^^,^^179^ Besides CLA, butyric acid, a SCFA produced through the metabolism of dairy products, was also shown to be protective in CRC.^180^^,^^181^ Lactic acid bacteria-fermented dairy products also exhibit potential anticancer properties, primarily through the modulation of gut microbial metabolism, attenuation of intestinal inflammation, and reduction in the absorption or bioavailability of carcinogens formed during high-temperature cooking. Unlike other dietary factors described above, the data are still inconclusive in terms of the involvement of dairy and dairy-based products in CRC, and more work in this field is warranted to affirm these associations.

#### Diet-microbe crosstalk in CRC pathogenesis

Although diet and gut microbiota alterations can independently influence CRC susceptibility in genetically predisposed individuals, their interaction often establishes an ecological niche that influences CRC development. Microbial metabolism of dietary constituents produces a diverse range of bioactive metabolites, such as SCFAs, long-chain fatty acids, and secondary bile acids, which collectively modulate epithelial turnover, DNA integrity, immune homeostasis, and oncogenic signaling pathways.^24-26^ Given the exceptionally high regenerative capacity of the intestinal epithelium and its continuous exposure to dietary inputs and microbial products, disruption of these regulatory processes increases vulnerability to malignant transformation. In this section, we explore how diet-driven changes in gut microbiota composition and metabolic activity shape epithelial metabolism, barrier function, inflammation, and genomic stability in CRC pathogenesis.

##### Diet-driven remodeling of microbial niches and microbial composition.

**Figure 2.**
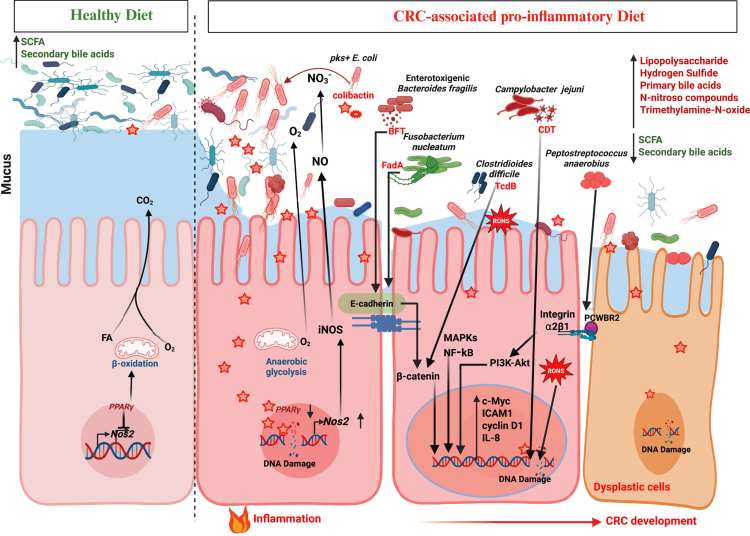
Diet-microbiome interactions shape host and microbial metabolism, mucosal inflammation, and CRC risk. Healthy dietary patterns promote SCFA production, fueling epithelial *β*-oxidation and activating PPARγ signaling, to maintain a hypoxic, anti-inflammatory, and homeostatic mucosal environment. In contrast, CRC-associated proinflammatory diets reshape microbial community structure and metabolic outputs, favoring pathobionts such as *pks*⁺ *Escherichia coli*, *Fusobacterium nucleatum*, enterotoxigenic *Bacteroides fragilis*, *Peptostreptococcus anaerobius*, *Campylobacter jejuni,* and *Clostridioides difficile*, thereby promoting CRC. These microbes produce genotoxins, including colibactin, CDT, and BFT, or express virulence factors such as FadA and TcdB, which alter epithelial proliferation and metabolism, induce RONS, and cause DNA damage. Diet- and inflammation-driven shifts in epithelial metabolism (e.g., altered *β*-oxidation) further reinforce dysbiosis and luminal nitrates (NO_3_^-^) availability, creating a permissive niche for pathobionts expansion, particularly of *pks*⁺ *E. coli*. Host‒microbe interactions additionally activate oncogenic and inflammatory signaling pathways, including NF-κB, MAPKs, PI3K-Akt, and *β*-catenin, which drive epithelial proliferation, chronic inflammation, genomic instability, and progression toward dysplasia and colorectal carcinogenesis. (Created with BioRender.com).

Dietary patterns profoundly influence the intestinal ecosystem by shaping inflammation, nutrient availability, and microbial metabolic niches, thereby modulating CRC risk. Western-style diets are consistently associated with chronic low-grade inflammation, microbial dysbiosis, and increased CRC incidence. Notably, such diets are linked to a higher prevalence of CRC tumors enriched for *pks⁺ E. coli*^182^ and induce systemic and intestinal inflammation,^183^ underscoring diet-induced inflammation as a key factor in shaping microbial niches by creating selective pressures that favor certain pathogenic microbes. Consistently, mucosal inflammation promotes the expansion of microbes with genotoxic potential, including *pks⁺ E. coli*, thereby accelerating colitis-associated carcinogenesis.^36^ Proinflammatory dietary regimens, particularly diets low in carbohydrates and fibers, induce mucosal inflammation and inducible nitric oxide synthase expression, resulting in increased production of host-derived RONS. These RONS contribute to CRC development by amplifying inflammation, altering epithelial stemness, increasing mutational burden, outpacing epithelial DNA repair processes, and enhancing susceptibility to malignant transformation.^18^^,^^39^^,^^184^ Oxidative DNA damage, particularly 8-oxoG, emerged as a key oncogenic lesion mediating RONS-driven genotoxicity in CRC.^39^ Beyond their direct genotoxic effects, inflammation-induced RONS also reprogram the metabolic landscape of the gut. Low-carbohydrate, low-fiber diets elevate the luminal nitrate levels in a PPARγ-dependent manner, generating a metabolic niche that selectively favors facultative anaerobes such as *pks*⁺ *E. coli* through nitrate-dependent respiration^23^^,^^185^^,^^186^ (Figure 2). In contrast, diets rich in fruits, vegetables, and whole grains that contain high fibers and polyphenols promote the increased abundance of beneficial taxa such as *Lactobacillus*, *Bifidobacterium*, *Faecalibacterium,* and *Ruminococcus*, while reducing *E. coli* and *Bacteroides uniformis*, reinforcing microbial functions that constrain inflammation, preserve epithelial integrity, and oppose tumorigenesis.^187^^,^^188-190^ Similarly, supplementing low-carbohydrate, low-fiber diets with the fermentable fiber inulin, which promotes *Lactobacillus* expansion and SCFA production,^191^ suppressed nitrate availability, and significantly reduced the growth, genotoxicity, and oncogenic activity of *pks⁺ E. coli*.^23^ Notably, oral tungsten supplementation that inhibits metalloenzymes required for nitrate respiration, selectively depletes *Enterobacteriaceae,*^192^^,^^193^ offering microbial metabolism as a tractable target for counteracting proinflammatory diet-induced dysbiosis and limiting the expansion of opportunistic genotoxic microbes that drive tumor initiation and progression.

Dietary components associated with red and processed meat intake further illustrate how nutrient availability intersects with microbial metabolic capacity to promote carcinogenesis. Nitrates and nitrites-rich meat-based products increase the abundance of *N*-nitroso compounds-producing bacteria, including *E. coli, Klebsiella, Proteus,* and *Pseudomonas*, taxa that typically remain at low abundance in the gut under normal homeostatic conditions.^194-196^ *N*-nitroso compounds generated through nitrosation within the gastrointestinal tract are potent carcinogens.^197^^,^^198^ In addition to *N*-nitroso compounds, heme and aromatic hydrocarbons derived from meat-based products can further perturb microbial composition by altering oxidative and nutritional landscape in inflammatory contexts, though their precise role in CRC remains unclear.^199^^,^^200^ Heme intake has been associated with increased abundances of *Enterobacteriaceae*, *Bacteroides*, *Helicobacter*, *Prevotella*, and *Akkermansia*, alongside with a reduced abundance of beneficial *Lactobacillus* spp.^200^ Emerging evidence also implicates trimethylamine-*N*-oxide (TMAO), a microbial metabolite derived from dietary choline, carnitine, and betaine, as another potential contributor to CRC. Elevated TMAO levels are associated with inflammation, oxidative and ER stress, metabolic dysfunction, and increased CRC risk.^201-204^ TMAO is generated by gut commensals through three distinct microbial pathways. The choline TMA-lyase (CutC/D complex) was first described in the anaerobic sulfate-reducing bacterium *Desulfovibrio desulfuricans*, which is also found in *Firmicutes, Actinobacteria*, and *Proteobacteria*, but not in *Bacteroidetes* in the human gut microbiome. The carnitine monooxygenase (CntA/B complex) is present in *Proteobacteria* and *Firmicutes,* while the members of the *Enterobacteriaceae* family use *γ*-butyrobetaine as a substrate to produce TMAO (the YeaW/X complex).^205-209^ These pathways highlight how specific microbial taxa and metabolic capacities interface with meat-derived dietary substrates to generate pro-carcinogenic metabolites.

High intake of sugars and SSBs further exacerbates diet-induced microbial dysbiosis, particularly under inflammatory conditions.^210-212^ Although the microbiota-dependent mechanisms linking sugar consumption to CRC remain incompletely defined, experimental studies demonstrate that SSBs worsen high-fat diet-induced colitis by altering gut microbial composition and promoting systemic inflammation.^210^ Artificial sweeteners, such as sucralose, similarly aggravate colitis and colitis-associated CRC in mouse models by inducing microbial dysbiosis.^213-215^ In the inflammation-associated CRC model induced by the mutagen azoxymethane (AOM) combined with dextran sodium sulfate (DSS), sucralose increased the abundance of *Firmicutes*, *Actinobacteria*, *Peptostreptococcus stomatis*, *Clostridium symbiosum*, and *Peptostreptococcus anaerobius*, while reducing *Proteobacteria*, a microbial shift linked to heightened inflammation and tumorigenesis.^213^

Alcohol consumption also reshapes microbial niches in ways that favor colorectal carcinogenesis. Chronic alcohol intake is associated with reduced microbial diversity and a shift toward dysbiosis, including a reduced fecal Firmicutes/Bacteroidetes ratio, which correlates with CRC development.^216^^,^^217^ Alcohol feeding also decreases dominant obligate anaerobes (*Bacteroides*, *Bifidobacterium*, *Ruminococcus*) while enriching mucosa-associated species such as *Streptococcus* and other opportunistic bacteria.^217^ Importantly, acetaldehyde, a primary carcinogenic metabolite of ethanol, is generated not only by host enzymes but also by gut microbes such as *Collinsella*, *Prevotella*, *Coriobacterium*, and *Bifidobacterium*, amplifying local carcinogenic exposure.^218^ Moreover, endogenous ethanol production through the metabolism of carbohydrates within the gut by *H. Pylori*, *Klebsiella pneumoniae*, *Enterobacter cloacae*, and *Escherichia coli*, and *Clostridium* *spp.*^219^^,^^220^ and its microbial conversion to acetaldehyde under aerobic gut conditions further promotes oxidative stress and DNA damage, both central to CRC pathogenesis.^221^

Additionally, host genetics also favors specific microbes in CRC pathogenesis.^222^ Lynch syndrome (LS) tumors, which arise from germline mutations in mismatch repair (MMR) genes, show enrichment of *F. nucleatum*, *pks⁺ E. coli*, but not *ETBF*, compared with sporadic MMR-proficient CRCs. Moreover, a SNP rs2355016 located in the intron of ATP-sensitive inward rectifier potassium channel 11 (KCNJ11) is associated with *F. nucleatum* abundance in CRC tumors. This SNP, rs2355016, led to lower KCNJ11 expression, increased Gal-GalNAc on CRC cells, and enhanced Fap2-mediated adhesion and invasion of *F. nucleatum*, promoting tumor proliferation.^223^ Dietary components and their metabolites further interact with host genetics by producing mutations or modulating the epigenome, thereby influencing gene expression programs that regulate immune responses, cellular proliferation, and DNA repair. A food-derived heterocyclic amine, 2-amino-1-methyl-6-phenylimidazo[4, 5-b]pyridine (PhIP), which selectively forms adducts on guanine residues, can induce allelic loss of MMR genes, resulting in MMR deficiency and hypermutagenesis.^224^ SCFAs derived from fiber fermentation, particularly butyrate, function as histone deacetylase (HDAC) inhibitors, while dietary methyl donors (e.g. folate, choline, betaine) modify DNA methylation patterns, altering chromatin accessibility in epithelial and immune cells. Together, these epigenetic effects may shape the mucosal microenvironment and modify host susceptibility to microbe-driven oncogenic pathways.

##### Dietary regulation of epithelial physiology and barrier function.

Another key determinant of pro-inflammatory diets in CRC pathogenesis is to disrupt epithelial integrity and barrier function. The colonic mucosa is protected by a two-layered mucus system, an inner sterile layer and an outer microbial layer, which together shield the epithelium from food and dietary toxins, luminal antigens, and microorganisms. Emerging evidence suggests that Western-style or fiber-free diets compromise this protective barrier by promoting the expansion of mucus-degrading microbes that erode the glycated mucus layer and/or by suppressing colonic Muc2 expression, thereby increasing epithelial vulnerability to microbe-induced colonic pathologies.^23^^,^^225^^,^^226^ Additionally, preclinical studies have demonstrated that dietary emulsifiers or food additives, such as carboxymethyl cellulose (CMC) and polysorbate 80, disrupt mucus architecture by altering mucus pore and thickness, triggering mucosal inflammation and barrier dysfunction.^227^^,^^228^ Barrier disruption allows pathogenic bacteria, such as *pks⁺* *E. coli*, to adhere closely to the epithelium, thereby amplifying their genotoxicity.^23^^,^^225^^,^^226^

Diet-induced shifts in microbial metabolism also compromise epithelial physiology. Meat-based diets favor the expansion of sulfate-reducing bacteria, resulting in elevated hydrogen sulfide (H_2_S), a microbial metabolite that is proinflammatory and cytotoxic at physiologically relevant concentrations.^229^^,^^230^ Increased abundance of H_2_S-producing taxa, including *Bilophila*, *Desulfovibrio*, *Bilophila wadsworthia,* and *Fusobacterium*, have been reported in colitis and CRC patients and are associated with epithelial DNA damage, hyperproliferation of colonic epithelial cells, and impaired barrier integrity.^231-233^ At millimolar concentration, H_2_S induces reductive stress and inhibits cytochrome oxidase, thereby suppressing oxidative phosphorylation and ATP generation in colonic epithelial cells.^234^ Similarly, red meat-derived heme iron increases the colonic abundance of mucus-degrading bacteria such as *Akkermansia muciniphila*, and promotes mucin breakdown and induces epithelial proliferation and hyperplasia.^235^^,^^236^ Similarly, diets high in simple sugars increase colitis susceptibility by altering microbial diversity and enriching mucolytic bacteria, leading to the disruption of mucosal barrier integrity.^237^ Excess sugar can additionally impair epithelial energy metabolism and proliferation independent of microbiota.^238^ Alcohol consumption similarly compromises intestinal barrier integrity by disrupting tight junction proteins such as Zonula Occludens-1 (ZO-1), increasing intestinal permeability, reducing microbial fermentation, altering epithelial metabolism and morphology, and facilitating uptake of luminal carcinogens. Collectively, these alterations promote hyperproliferation of epithelial cells and CRC risk.^239^^,^^240^

In contrast, diets rich in fruits and vegetables support epithelial health and barrier resilience. High intake of plant-based foods is associated with increased abundance of beneficial taxa, including *Lactobacillus*, *Bifidobacterium*, *Faecalibacterium,* and *Ruminococcus*, which in turn contribute to enhanced barrier function, immune regulation, and epithelial metabolic homeostasis. These effects are largely mediated by dietary fibers, which fuel the production of SCFAs that reinforce epithelial barrier integrity and exert anti-inflammatory and anti-neoplastic effects.^241-247^

##### Dietary regulation of microbial virulence in CRC.

Dietary substrates and their microbial metabolites can directly modulate microbial virulence by affecting interbacterial competition, epithelial adherence, and virulence gene expression. Inflammation has been shown to upregulate virulence genes such as the *pks* island in *E. coli*, whereas anti-inflammatory interventions like anti-TNF therapy suppress their expression,^248^ highlighting that inflammatory factors, including diets, can modulate the virulence of CRC-associated microbes to influence colorectal carcinogenesis. Epidemiological evidence indicates that high dietary fiber intake correlates with lower intratumoral loads of *pks⁺ E. coli* and *F. nucleatum*, and reduced CRC risk. Consistent with these findings, we and others have reported the protective effect of inulin on CRC development.^23^^,^^191^^,^^249^ Our study demonstrated that supplementing a low-carbohydrate, low-fiber diet with inulin protects *Il10*^*⁻/⁻*^ mice from *pks⁺ E. coli*-induced tumorigenesis.^23^ However, an *in vitro* bacterial culture study shows that inulin can upregulate phosphopantetheinyl transferase *clbA*, a key enzyme for colibactin synthesis,^250^ and consistently in *Apc*^*Min/+*^ mice, dietary inulin increased colibactin-dependent genotoxicity and tumor formation.^251^ These apparent discrepancies might be explained by differences in mouse models, gut microbial composition, CRC stage, and fiber dose,^249^ emphasizing that the impact of dietary fiber on CRC depends on microbial composition, host physiology or genetics, and tumorigenic context. A recent study has reported that prudent diets rich in whole grains and dietary fiber can reduce the risk of *F. nucleatum*-positive CRC, but not *F. nucleatum*-negative CRC, supporting a potential role of specific microbes in mediating the association between diet and colorectal neoplasms.^252^

Other dietary factors also influence microbial toxin production. High iron intake suppresses *clbA* transcription and colibactin synthesis via a noncanonical Fur/RyhB pathway,^253^ whereas spermidine, which is abundant in legumes, soy, and cereals, enhances colibactin production.^254^ Enterotoxigenic *Bacteroides fragilis* toxin (BFT) expression is inhibited by free glucose, galactose, and plant-derived fermentable carbohydrates, but can be upregulated by oxygen and heat stress,^76^ although its influence on tumorigenesis requires further investigation. Similarly, fusobacterial adhesins and virulence factors, such as Fap2 and FadA, can be downregulated by aspirin *in vitro,*^255^ suggesting that diet or pharmacological agents may modulate fusobacterial pathogenicity. This topic of dietary modulation of microbial virulence is especially relevant given the widespread use of *pks⁺ E. coli* Nissle 1917 (*EcN*) as a probiotic, a strain capable of producing colibactin^256^ and causes DNA damage and mutations.^257^ Although EcN exhibits lower genotoxicity and reduced epithelial adhesion compared with CRC-associated *pks⁺ E. coli* strains,^60^ its inherent genotoxic potential may be enhanced under certain microenvironmental conditions. Therefore, further studies examining *EcN* under diverse dietary conditions are needed to clarify its potential impact on CRC risk.

Collectively, these findings highlight critical mechanistic links among dietary factors, the gut microbial community, host mucosal physiology, and CRC development. While it is now evident that dietary factors can modulate both the composition and functional activity of the gut microbiota implicated in CRC pathogenesis (Table 1), most existing evidence remains largely correlative and lacks functional validation, limiting the ability to establish definitive causal relationships between diet-induced microbial alterations and CRC pathogenesis.

**Table 1.** Selected dietary intervention studies in CRC that consider the microbiome.

Dietary intervention	Dietary regimen	Human/animal model	Microbial abundance	CRC-associated biomarkers	CRC/tumors development	References
High-fat diet (43.1% fat)	12 weeks (*Apc*^min/+^ mice); 22 weeks (AOM-treated conventional C57BL/6 mice)	Mouse	*Alistipes* spp. Marseille-P5997 ↑; *Alistipes* sp.5CPEGH6 ↑; *Parabacteroides distasonis* ↓ in feces	Impaired gut barrier function; Colonic cell proliferation ↑; Oncogenic genes expression ↑	↑	[264]
High-fiber diet (20% soluble and 20% insoluble fiber)	12 weeks (*Apc*^min/+^ mice); 22 weeks (AOM-treated conventional C57BL/6 mice)	Mouse	*Bacteroides uniformis* ↑; *Bifidobacterium pseudolongum* ↓ in feces	Serum Bile acids ↑; Inosine ↓; Fecal butyrate ↑	↑	[265]
Pesco-vegetarian diet	3 months (*Apc*-mutated or AOM-induced PIRC rats)	Rat	*Prevotellaceae NK3B31*, *Lachnospiraceae NK4A136*, *Lachnospiraceae UCG-001*, *Papillibacter*, *Paraprevotella*, *Enterorhabdus*, and *Parvibacter* in feces were associated with the Pesco-vegetarian dietary group	Aberrant crypt foci ↓; Apoptosis in tumors and normal mucosa ↑; Myeloperoxidase activity ↓; TNF-*α* ↓; Gut permeability ↓; TBARS and 4-HNE ↓	↓	[266]
Fasting-mimicking diet (FMD)	Six FMD cycles (AOM-DSS-treated C57BL/6 mice); two FMD cycles (orthotopic MC38 tumor model)	Mouse	*Bifidobacterium pseudolongum* and *Lactobacillus reuteri* ↑ in feces	Tumor load ↓; Tissue-resident memory CD8^+^ T-cell ↑; L-arginine ↑; Anti-CTLA-4 efficacy ↑	↓	[43]
Ketogenic diet	5 weeks (AOM-DSS-treated C57BL/6 J mice)	Mouse	*Phocea* spp., *Faecalitalea* spp., *Akkermansia* spp., *Intestimonas* spp.*, Lachnoclostridium* spp., and *Bilophila* spp. ↑; *Barnesiella* spp.*, Eisenbergiella* spp.*, Ruminococcus 1* and *2* spp., *Bifidobacterium* spp., and *Turicibacter* spp. ↓ in feces	Colonic tumor burden ↓; IL17-producing T cells ↓; IL-6, STAT3, IFNγ, and TNFα ↓; Natural killer T cells and type 3 innate lymphoid cells ↑; Plasma *β*-hydroxybutyrate ↑	↓	[26]
High-fiber diet (20% insoluble fermentable fiber and 50% raw potato starch)	12 weeks (TS4Cre × cAPC^lox468^ mice)	Mouse	*Bifidobacterium, Lachnospiraceae_unclassified,* and *Anaerostipes* ↑ in feces	SCFAs ↑; GPR109a expression ↑	↓	[267]
Low-carbohydrate low-fiber diet (6.8% carbohydrate)	8 weeks (*E. coli* NC101-infected *Il10*^*-/-*^ mice)	Mouse	*Enterobacteriaceae* ↑; *E. coli* NC101 ↑; *Bifidobacterium*, *Turicibacter,* and *Marvinbryantia* ↓ in feces	Mucosal inflammation and luminal nitrates ↑; colonic PPARγ signaling ↓	↑	[23]
Diet supplemented with Proanthocyanidins from black soybean seed coat	7 weeks (*Apc^min/+^* mice)	Mouse	*Paramuribaculum, Alistipes, Desulfovibrio,* and *Tyzzerella* ↑; *Parabacteroides* ↓ in feces	Intestinal polyps ↓; proliferating cell nuclear antigen (PCNA) and *β* catenin ↓; SCFAs ↑	↓	[268]
Diet with Rice Bran	15 weeks (AOM-DSS-treated Balb/c mice)	Mouse	*Clostridiales*, *Blautia*, *Roseburia* ↑ in the gut mucosa	Neoplastic lesion size ↓; Mucosal and systemic inflammation ↓	↓	[269]
Diet containing freeze-dried baby spinach (10% w/w)	26 weeks (F344/NTac-*Apc*^*am1137*^) rats	Rat	*Proteobacteria* and *Tenericutes* ↓; *Verrucomicrobia* ↑ in gut contents	Intestinal polyps ↓; Linoleate and butanoate metabolism ↑	↓	[270]
Diet containing long-chain inulin (10%)	17 weeks (AOM-treated Sprague–Dawley rats)	Rat	*Lactobacillus spp.* and *Bifidobacteria* ↑; *Escherichia coli and Salmonella enterica* serovar Typhi ↓ in cecal contents	aberrant crypt foci ↓; Phenol, *p*-cresol, and indole ↓	↓	[271]
Sucralose (1.5 mg/ml)	11 weeks in drinking water (AOM-DSS-treated C57BL/6 mice)	Mouse	*Firmicures*, *Clostridium symbiosum*, and *Peptostreptococcus anaerobius* ↑; *Solobacterium moorei* and *Bifidobacteria* ↓ in feces	Colonic tumors ↑; gut permeability ↑; TNFα, TLR4, and Myd88 ↑; IL-10 and IκBα ↓; VEGF and PCNA ↑	↑	[213]
Diet containing inulin (5 and 15%)	8 weeks (AOM-treated *A/J ^Min/+^* mice)	Mouse	*Bacteroidaceae*, *S24-7*, *Erysipelotrichaceae,* and *Alcaligenaceae* ↑ in cecal contents	Colonic tumors ↓;	↓	[249]
Diet containing inulin (10%)	4 weeks (*E. coli* NC101-infected *Apc^Min/+^* mice)	Mouse	*E. coli* NC101 ↑ in feces	Intestinal tumors ↑; Double-stranded breaks ↑	↑	[251]
Green leafy vegetables (GLVs)	12 weeks of GLVs intervention in individuals consuming atleast five servings of red meat per week	Randomized controlled Meat and Three Greens (M3G) Feasibility human trial ]	Change in fecal *Proteobacteria* in females and *Bifidobacterium* and *Bacteroidaceae* in men was associated with serum zonulin; *Faecalibacterium* and *Faecalibacterium prausnitzii* were inversely associated with fecal 8OHdG	Plasma and fecal 8-hydroxy-2'-deoxyguanosine (8OHdG) ↓; TNFα ↓	Not directly evaluated	[272,273]
Dietary inulin supplementation ]	24 g of inulin or placebo daily for 3 weeks	Randomized, double-blind, crossover human trial	*Bacteroides fragilis* ↓ in feces	Butyrate ↑; pH ↓; Secondary bile acids ↓; Mucosal inflammation ↓	Not directly evaluated	[274]
Prebiotic diet with canned beans	1 cup/d of canned beans (contains 16 g dietary fiber and 14 g protein) for 8 weeks to obese patients with a history of colorectal neoplasia	Randomized BE GONE dietary intervention human trial	*Faecalibacterium, Eubacterium,* and *Bifidobacterium* ↑ in feces	pipecolic acid ↑; Indole ↓; Fibroblast-growth factor-19 ↑; Immuno-oncology cytokines ↓	Not directly evaluated	[275]
High red and processed meat intake vs. Mycoprotein intake	240 g/d red and processed meat for 2 weeks, with crossover to 2 weeks 240 g/d mycoprotein	Investigator-blind, randomized, crossover dietary intervention human trial	Fecal *Oscillobacter* and *Alistipes* ↑ in meat diet group; *Lactobacilli*, *Roseburia,* and *Akkermansia* ↑ in Mycoprotein group	Fecal genotoxicity and nitroso compound ↑ in meat diet group, but ↓ in Mycoprotein group; SCFAs ↑ in Mycoprotein group	Not directly evaluated	[276]
Rice bran dietary intervention	30 g of rice bran over 24 h for 24 weeks (Subjects with a high risk of CRC)	Double-blind, randomized, placebo-controlled human trial	*Firmicutes*, *Lactobacillus* and *Bifidobacteria* ↑; *Firmicutes*/*Bacteroidetes* ratio ↑; *Prevotella_9* ↑ in feces	Not directly evaluated	Not directly evaluated	[277]
Cooked red meat (HRM) diet without or with butyrylated high-amylose maize starch (HRM + HAMSB)	300 g/d of cooked red meat without or with 40 g/d of HAMSB for 4 weeks	Randomized cross-over human trial	*Clostridium coccoides*, *Clostridium leptum*, *Lactobacillus* spp., *Parabacteroides,* and *Ruminococcus bromii* ↑, but *Ruminococcus torques*, *Ruminococcus gnavus*, and *Escherichia coli* ↓ in feces of HRM + HAMSB group	Rectal O(6)-methyl-2-deoxyguanosine (O(6)MeG) adduct levels ↑ in HRM, but ↓ in HRM + HAMSB group; SCFAs ↑ in HRM + HAMSB group	Not directly evaluated	[278]
High-fiber, low-fat African-style diet	2 weeks food exchange (African Americans were fed a high-fiber, low-fat African-style diet)	Dietary exchange human trial	Shift from correlations between fecal *Bacteroides* and potential butyrate-producing groups (*Roseburia intestinalis* et rel. and *Clostridium symbiosum* et rel.) towards stronger co-occurrence patterns, including Firmicutes that are typically associated with complex carbohydrate fermentation	Colonic mucosal inflammation ↓; Proliferation biomarker Ki67 ↓ in colonic mucosa; Saccharolytic fermentation and butyrogenesis ↑; Secondary bile acid ↓;	Not directly evaluated	[183]

## Conclusions and perspectives

The gut microbiome holds great promise as a source of biomarkers for CRC diagnosis, prognosis, and therapeutic stratification, with taxa such as colibactin-producing *E. coli*, *F. nucleatum*, and enterotoxigenic *B. fragilis* that are linked to tumor initiation and progression. Microbiota profiling from fecal samples offers a noninvasive tool for early detection and staging, and microbial signatures can predict treatment response, allowing identification of patients at high risk of recurrence. Integrating microbiota analysis with machine learning and artificial intelligence could further enhance predictive accuracy and accelerate clinical translation. Beyond their diagnostic potential, strategies that manipulate the microbiome, such as dietary interventions, probiotics, or fecal microbiota transplantation, offer opportunities to prevent CRC or enhance therapeutic outcomes by modulating immune responses and reducing inflammation.^258-263^ However, several challenges hinder clinical implementation, including high interindividual variability, lack of standardized sampling and analysis protocols, incomplete understanding of host–microbiota–immune interactions, safety concerns–particularly in immunocompromised patients, and limited evidence from large, randomized controlled trials. Critical knowledge gaps remain regarding the exposure thresholds and duration of colonization by genotoxic microbes necessary to drive oncogenic mutations, the host and dietary factors that determine susceptibility, and the potential synergistic interactions among multiple CRC-associated microbes. Furthermore, while experimental studies highlight the influence of diet on microbial genotoxicity, translating these findings to human populations is complex, and changes in microbial composition alone cannot be assumed causal without direct mechanistic validation. Addressing these challenges will require multidisciplinary efforts, development of precision microbiome profiling tools, and rigorous translational studies to establish safe and effective microbiota-based strategies for CRC prevention and therapy.
